# Factors influencing the community participation approaches used in *Aedes* mosquito management in the Torres Strait, Australia

**DOI:** 10.1186/s12889-023-16942-8

**Published:** 2023-10-12

**Authors:** Tammy Allen, Alan Crouch, Tanya L. Russell, Stephanie M. Topp

**Affiliations:** 1 College of Public Health, Medical and Veterinary Sciences, James Cook University, Queensland, Australia; 2 Department of Rural Health, University of Melbourne, Victoria, Australia; 3 Australian Institute of Tropical Health and Medicine, James Cook University, Queensland, Australia

**Keywords:** *Aedes* mosquito management, Community participation, High-income countries

## Abstract

**Background:**

*Aedes*-borne disease risk is increasing in tropical and sub-tropical regions across the globe. While *Aedes*-borne disease continues to disproportionally affect low- and middle-income countries, parts of high-income countries, such as the Torres Strait region in Australia are also at risk. The Torres Strait is a group of islands located between Cape York Peninsula in far north Queensland, Australia and Papua New Guinea. The Torres Strait has both *Aedes albopictus and Aedes aegypti* and is close to Papua New Guinea where dengue fever is endemic. Managing *Aedes-borne* disease risk requires a range of strategies, including community participation. Existing research shows that high-income countries tend to favour government-led (top-down) informing approaches when engaging communities in *Aedes* mosquito management. Little is known about the factors that influence the choice of community participation approaches in *Aedes* mosquito management particularly in a high-income country setting, such as Australia. This research contributes to filling this knowledge gap by exploring the community participation approaches used in *Aedes* mosquito management and the factors influencing these choices in the Torres Strait.

**Methods:**

16 semi-structured interviews were conducted with local government and state government agencies working in *Aedes* mosquito management in the Torres Strait. Six key mosquito management plans and policies were also reviewed. Thematic analysis was used to identify, analyse and attribute meaning from the data collected.

**Results:**

A range of community participation approaches were used within the two main *Aedes* mosquito management programs (*Aedes albopictus* Elimination Program and the Torres Strait Island Regional Council, Environmental Health Program) in the Torres Strait. These approaches included door-to-door inspections, awareness raising strategies, and community clean-up events. Approaches were chosen for reasons related to regulations, attitude and beliefs, and resourcing.

**Conclusions:**

This study revealed the use of both top-down and bottom-up approaches to engaging the community in *Aedes* mosquito management in the Torres Strait. These findings contribute to a better understanding of why bottom-up approaches are used, which is valuable for shaping future policy decisions. This study also provides suggestions on ways to enhance community participation in the Torres Strait, which could also be considered in other similar tropical regions.

## Background

Over the last few decades, there has been an increase in the incidence of *Aedes* mosquito-borne diseases such as dengue fever, with an estimated 100–400 million global infections occurring each year [1]. Disease transmission risk is highest in tropical and sub-tropical regions where climate, social and environmental conditions support prolific *Aedes* mosquito populations [2]. With an estimated 87% of countries world-wide (n = 215 countries) having favourable conditions for *Aedes aegypti* and/or *Aedes albopictus* to establish, there is mounting concern that *Aedes* species, particularly *Ae. albopictus*, will spread to new tropical and sub-tropical regions [2–4]. While *Aedes*-borne disease disproportionally affects low- and middle-income countries, high-income countries, characterized by advanced healthcare systems and infrastructures, are not immune to the threat of *Aedes*-borne disease. For example, parts of Europe, the United States and Australia have experienced *Aedes*-borne disease outbreaks, with the risk of these outbreaks increasing with global travel, trade, urbanization, and climate change [3, 5–7].

### ***Aedes***-borne Disease risk in Australia

Australia has a history of *Aedes*-borne disease outbreaks, specifically dengue fever, dating back to the late nineteenth century [8]. During the 1950s, the introduction of reticulated water systems largely led to the disappearance of *Ae. aegypti* in parts of Australia. This innovation eliminated the need for rainwater tanks, consequently reducing breeding opportunities for *Ae. aegypti*. Despite this, *Ae. aegypti* remained established in north Queensland, and has been found as far south as Gin Gin, South-East Queensland, and in Tennant Creek, Northern Territory [9–11].

Since the early 2000’s, north Queensland has experienced several large dengue fever outbreaks including a multi-city outbreak in 2003/2004 (900 confirmed cases) and an outbreak in Cairns in 2008/2009 (938 confirmed cases) [12, 13]. However, over the last decade, *Aedes*-borne disease risk has reduced in parts of north Queensland including the regional cities of Townsville and Cairns which can be directly attributed to the World Mosquito Program (WMP). Over the last decade, the WMP has successfully introduced *Wolbachia* infected *Ae. aegypti* into these communities. With *Wolbachia* acting as a virus blocker in the mosquito, this method has so far shown to be successful in reducing *Aedes*-borne disease transmission risk [14].

Despite this development, *Aedes*-borne disease risk remains in parts of Queensland. There is an ongoing risk of *Ae. albopictus* incursion from the Torres Strait to mainland Australia, via ports/travel. If *Ae. albopictus* was to enter Australia, it could establish in both tropical and sub-tropical regions [15, 16]. Global travel, favourable social and environmental conditions, including a growing number of households with rain-water tanks, and low community immunity could lead to sporadic or annual dengue fever outbreaks in large cities, such as Brisbane [4, 17].

**Fig. 1:**
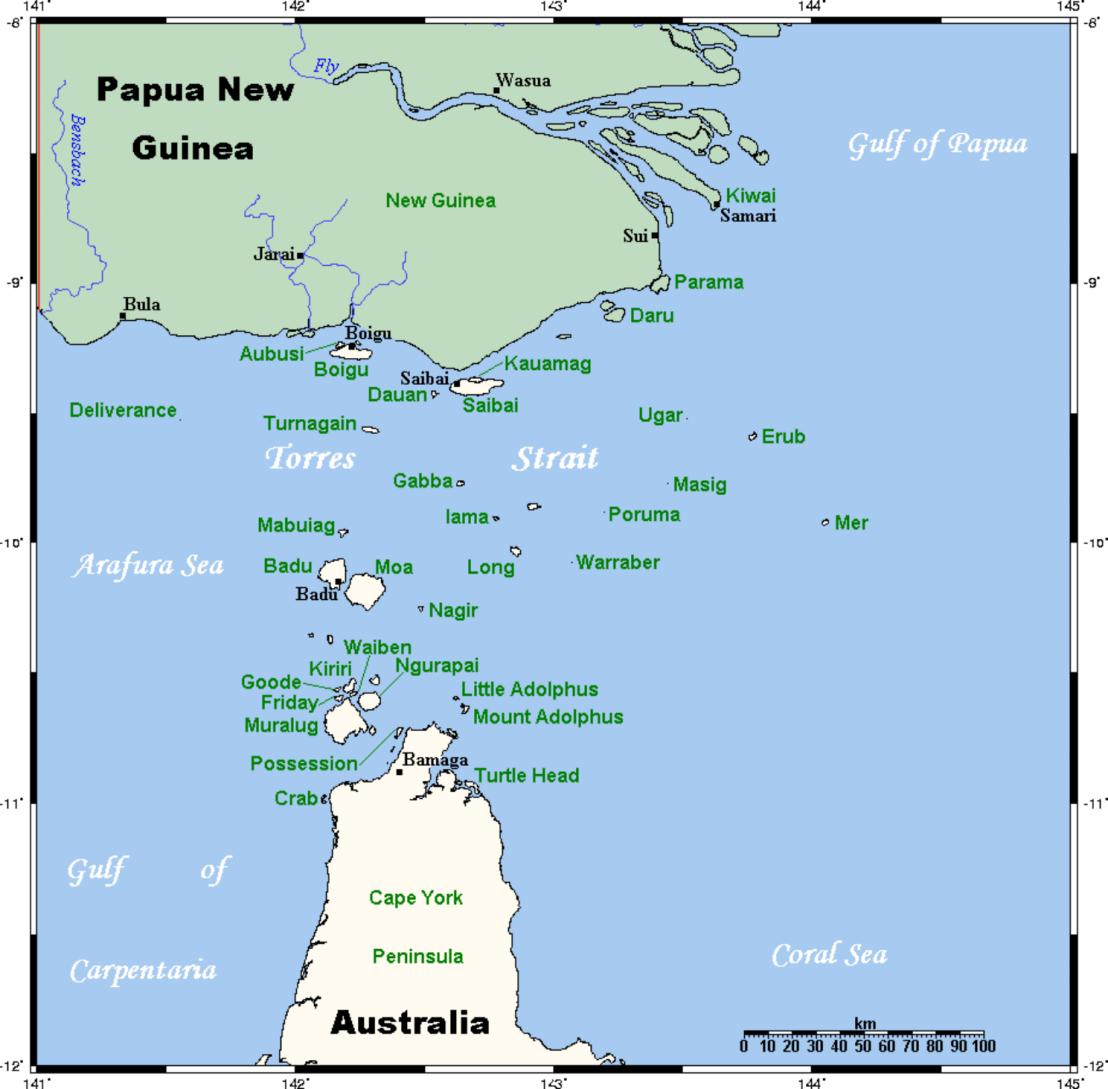
Map of the Torres Strait. Ref: https://commons.wikimedia.org/wiki/File:TorresStraitIslandsMap.png

Another region in Queensland at risk of *Aedes*-borne disease is the Torres Strait. The Torres Strait is located between Cape York Peninsula, in far north Queensland and Papua New Guinea. This unique, geographical region comprises eighteen inhabited islands, including two “inner” islands (due to their close proximity to mainland Australia), namely Waibene (hereafter referred to as Thursday Island) and Ngurupai (hereafter referred to as Horn Island) and sixteen “outer” islands [18] (Fig. 1). Approximately 7,500 individuals live in the Torres Strait, with 75% identifying as Torres Strait Islander, and 22% identifying as both Aboriginal and Torres Strait Islander [19].

While both *Ae. aegypti* and *Ae. albopictus* co-exist in the Torres Strait, as of 2016, *Ae. albopictus* was confirmed as the dominant *Aedes* species in this region [20]. *Aedes*-borne disease transmission risk in the Torres Strait is heightened due to population mobility between the Torres Strait Islands, as well as cross-border to Papua New Guinea, where dengue fever is endemic [21]. Consequently, the Torres Strait has a history of dengue fever outbreaks, for example in 2003–2004 an outbreak resulted in 176 confirmed cases and two deaths, and in 2016 an outbreak across multiple islands resulted in 26 confirmed cases [22]. The Torres Strait is also at risk of climate impacts (e.g. changes to temperature and rainfall), which could potentially increase mosquito-borne disease risk [23].

### ***Aedes*** mosquito management in the Torres Strait

There are currently two main *Aedes* mosquito management programs running in the Torres Strait:

1) Torres Strait Island Regional Council (local government), Environmental Health Program, which comprises environmental health workers responsible for conducting routine vector control activities on the outer islands [24].

2) Queensland Health (state government) *Aedes albopictus* Eradication/Control Program, which utilizes a fly-in, fly-out workforce of vector control officers from the public health unit in Cairns, who travel to Thursday Island and Horn Island. This program’s primary goal is to suppress *Ae. albopictus* on these two inner islands, using intensive vector control measures [16].

### Community participation in *Aedes* Mosquito Management

Community participation in *Aedes* mosquito management is recognised to be important, given the *Aedes* species, particularly *Ae. aegypti*, lives and breeds close to its food source – humans [25]. Community participation may include contributing to source reduction efforts, helping monitor mosquito populations, assisting in disease surveillance or in implementing novel population suppression or replacement strategies such as those conducted by the World Mosquito Program [26–29].

The World Health Organization emphasizes the importance of using participatory (often referred to as ‘bottom-up’) approaches to foster community ownership and promote sustainable *Aedes* mosquito management practices [25]. A review examining the community participation approaches used in *Aedes* mosquito management specifically in high-income countries, found an overall preference for more centralized, government-led (often referred to as top-down) informing approaches [29]. Such choices are of interest, as although top-down approaches can be effective, particularly when lead by a strong, centralized government, they can also be resource-intensive, expensive and difficult to sustain [30]. Hence, top-down approaches can also contribute to community apathy and lead to unrealistic expectations of the government’s role in *Aedes* mosquito management [30]. The reasons for favouring top-down approaches in high-income countries are not clear. To help build an understanding of the challenges and opportunities in strengthening community participation, it is essential to explore the factors that influence the choice of community participation approaches used in *Aedes* mosquito management in high-income countries such as Australia.

This qualitative case study had two objectives:

1. To understand the types of community participation approaches used in *Aedes* mosquito management in the Torres Strait, Australia.
2. To explore the factors that influence the choice of the community participation approaches used in *Aedes* mosquito management in the Torres Strait, Australia.

## Methods

### Study design

A case study design incorporating multiple qualitative methods was used in response to the study questions. We report here on the findings specifically related to the Torres Strait, Queensland, Australia. The case unit was defined by the geography of the Torres Strait (18 inhabited islands) and the *Aedes* mosquito management programs implemented within this region. Case boundaries were extended as necessary to account for programs funded or administrated at the state and federal level.

### Data collection

Data collection, between August 2019 and July 2022, consisted of two stages. Stage One was a document analysis of current and historical *Aedes* mosquito management plans (N = 6) focused on the Torres Strait region. These documents were obtained from state and local government websites and key informants. Stage Two involved conducting semi-structured interviews with key informants. Purposive and snowball sampling was used, drawing on investigator expertise and public access information, to identify suitable participants. Interviews participants needed to be currently or previously employed in *Aedes* mosquito management in the Torres Strait.

A total of 16 semi-structured interviews were conducted, comprising nine state government (Queensland Health) current/previous employees and seven local government (Torres Strait Island Regional Council) current/previous employees. Interviews spanned those with responsibilities for general environmental health inclusive of vector control, to specialized vector control. Twelve interviews were conducted face-to-face in Cairns or Thursday Island in the Torres Strait. Four interviews were conducted via video conference due to local COVID-19 travel protocols restricting travel during the data collection period.

### Data analysis

Thematic analysis was used to identify, analyse and attribute meaning from the data collected. Each interview was transcribed verbatim and imported into NVivo12+, with a selection of transcripts sent back to participants for checking. The data was initially coded inductively to ensure attention was paid to key issues within participant accounts. Subsequent rounds of deductive analysis drew on the IAP2 Public Participation Spectrum© [31] and Community Empowerment Domains [32] to help categorise the range of community participation approaches chosen (Objective One). Scott’s Institutional Analysis Theory [33] was used to help make sense of the regulatory, cognitive and resource factors driving the choice of approaches in the different programs and at different times (Objective Two). This combination of inductive and deductive coding ensured analysis remained linked to the data, as well as responding to the key research objectives outlined.

### Ethics statement

The study was approved by the Townsville Hospital and Health Service HREC Human Research Ethics Committee, Australia (HREC/2019/QTHS/53,053). Interviews and subsequent analysis followed the relevant guidelines and regulations as stipulated in the approval. Informed consent was obtained from all the participants, who were provided with a participant information sheet and consent form, which the participants signed. Participation in the study was voluntary and confidential. Standards for Reporting Qualitative Research were used as a guide for reporting on this research [34]. In consideration of culturally safe protocols for researching Aboriginal and Torres Strait Islander people [35] the draft research proposal was presented and discussed with the Torres Strait Island Regional Council prior to the project commencing.

## Results

The findings are organized into two sections. First, we describe the community participation approaches used in the two main *Aedes* mosquito management programs in the Torres Strait – the Torres Strait Island Regional Council, Environmental Health Program and the Queensland Health, *Aedes albopictus* Eradication/Control Program (Objective One).

Second, we examine the key factors influencing the choice of community participation approaches used in these programs (Objective Two). The key factors are described under the following themes – regulatory (laws and regulations that shape community participation approaches); cognitive (agency’s attitudes and beliefs towards engaging the community); and human and other resources. Quotes were chosen to represent the key theme discussed, and are attributed by the agency – local government, Torres Strait Island Regional Council (TSIRC) or state government, Queensland Health (QH) and a sequential ID.

### Community participation approaches used in *Aedes* mosquito management

#### Torres Strait Island Regional Council, Environmental Health Program

The Torres Strait Island Regional Council, Environmental Health Program commenced in the early 2000s, with the aim of employing local environmental health workers (EHWs) to address environmental health issues including *Aedes* mosquito management on each of the sixteen inhabited islands of the Torres Strait [36]. Although funded by state government (Queensland Health), the EHWs were managed by separate local government councils on each of the islands. In 2008, a Local Government Reform Implementation Regulation was introduced [37] leading to the merging of outer island councils into a single entity, known as the Torres Strait Island Regional Council (TSIRC). Consequently, the EHWs were managed under the one TSIRC, Environmental Health Program [38]. At the time of data collection, there were sixteen EHWs located across the outer islands of the Torres Strait. The EHW’s primary focus in *Aedes* mosquito management was conducting house to house source reduction strategies, spraying and mosquito surveillance [39].

The main focus of engaging the community in *Aedes* mosquito management on the outer islands was to increase awareness of dengue fever, including promoting protective and preventative behaviours such getting rid of potential larval habitats, screening homes, and wearing insect repellent; and to actively encourage community involvement in source reduction activities [39].

During the interviews, the EHWs elaborated on a range of community participation approaches used, which were similar across the different islands. Firstly, the EHWs described visiting schools to develop children’s knowledge of mosquito-borne diseases and mosquito breeding. This included conducting presentations, for example, on the characteristics of *Aedes* species. Several of the EHWs noted the importance of engaging children as a catalyst for spreading dengue fever awareness to other family members.

*“You find if you do it at school, then they tell everyone else, you know? They’re really good at reminding adults. When I do the clean-ups, the majority of them are the kids.”(TSIRC,1)*.

One EHW described conducting community presentations and disseminating resources to promote dengue fever prevention, using a mosquito identification book to help the community understand the different types of mosquito species that can carry disease.

“When we do talks or whatever, we pass out information. What I found some people were really interested in was, there’s this book… mosquito identification. They learned *about* a bunch of mosquitoes. The kids, they enjoy that as well” (TSIRC,1).

All the EHWs described engaging the community in clean-up events, to promote active participation in reducing micro and macro waste. These events also provided the opportunity for the EHWs to show the community potential larval habitats.

*“…When I do the clean-up, we talk about why we don’t throw rubbish. We talk to the people and explain to them like even a small bottle top, that can breed mosquitoes. That and actually showing them the pupae and stuff like that.”(TSIRC,1)*.

Community clean-ups were also supported by a weekly council-run, kerb-side rubbish collection, an additional strategy to the normal household waste disposal service provided be the council.

*“…we try to encourage families to have regular clean-ups. The council can collect rubbish once a week, but any other rubbish they put up besides the domestic rubbish, the council are able to collect it once a week. So, no one really should be having heaps of rubbish around their houses.” (TSIRC,1)*.

Some of the EHWs described how they spent time educating residents on where mosquitoes can breed as part of door-to-door inspections, to increase awareness of mosquito breeding in and around their homes.

*“I explain to them that the eggs can stay there years and years and months, just waiting for one good drip of water and maybe go again. I tell them to always wipe the surface of the container to get rid of the eggs and all that. Also give them flyers that says clean around the house and what you’re meant to look for.”(TSIRC,2)*.

The TSIRC Environmental Health Program was also found to have elements of empowering approaches, such as using local leadership (through employing local EHWs), drawing on local resources and knowledge, and actively encouraging community involvement (as previously described). The EHWs also described autonomy with developing and implementing local, culturally appropriate strategies to promote participation in *Aedes* mosquito management on each of the islands.

### Queensland Health, *Aedes albopictus* Eradication/Control Program

The *Aedes albopictus* Eradication/Control Program (AAEP) is a federally funded, state government resourced program established in 2005, in response to the discovery of *Ae. albopictus* in the Torres Strait. The initial aim of the AAEP was to eliminate the *Ae. albopictus* from the Torres Strait. To achieve this, nine vector control staff from the public health unit in Cairns, travelled approximately 900 km from Cairns to the Torres Strait, to conduct source reduction strategies on multiple islands over a two-week period, several times a year [16]. During this initial period (hereafter referred to as Phase One), a range of community participation strategies were used with the primary purpose to gain community support for the AAEP, and to encourage the local Torres Strait Islander community’s involvement in source reduction strategies. Most of the engagement strategies conducted by the AAEP, were in conjunction with the local EHWs stationed on each island. Engagement with the EHWs helped establish a direct link with the local communities particularly given the AAEP staff were conducting vector control in private properties.

*“Generally the community, also have a EHW who you would have to link up with, because they are the ones to deal with the mosquito elements of the community. If they are on the ground, that is very helpful. They are also kind of coordinators, so that if there is an outbreak on one island, there’s one or more EHWs they bring in some from nearby islands.”(QH,1)*.

The AAEP staff also collaborated with the EHWs to conduct community education, through door-to-door inspections and school visits. Key source reduction messages such as getting rid of, emptying or covering potential larval habitats were also promoted through the local radio and newspaper advertisements [16].

By 2008, the logistical and resourcing challenges from regular and intensive vector control operations across sixteen islands, using a fly-in, fly-out workforce, became too significant to successfully eliminate *Ae. albopictus* from the Torres Strait. A decision was made to cease operating on the outer islands and redirect efforts to the two inner islands of Thursday Island, (consisting of 65% of the Torres Strait population) [19] and Horn Island, the main commercial and transport hubs linking the Torres Strait to mainland Australia [16].

The main aim of this new phase (hereafter referred to as Phase Two) was to reduce the risk of the *Ae. albopictus* mosquito entering mainland Australia. A *cordon sanitaire* approach was adopted that focused on preventing *Ae. albopictus* from establishing on the two islands. However in 2010, *Ae. albopictus* was discovered on both islands, so the approach changed to suppressing *Ae. albopictus* using additional vector control strategies including intensive harbourage spraying and lethal tyre piles [16].

Phase Two community engagement was focused on liaising with the local council, and community leaders to gain approval to run the AAEP on the two islands. There was generally little engagement with local environmental health staff, and community members were informed of the AAEP operations through a local newspaper announcement.

### Factors influencing the choice of community participation approaches used in *Aedes* mosquito management

Three key sets of factors were found to influence the community participation approaches used in the TSIRC, Environmental Health Program and the AAEP (Phase One and Phase Two); namely regulatory, cognitive, and resource factors.

### Factor one: Regulatory factors influencing community participation approaches

The community participation approaches used by both the AAEP staff and the TSIRC Environmental Health Program EHWs were shaped by Queensland legislation [40, 41]. This legislation outlined the role of state government (Queensland Health), local government (TSIRC Environmental Health Program) and community members in *Aedes* mosquito management. Under the Queensland Public Health Act (2005), mosquito breeding habitats are considered a public health risk, with the Queensland Public Health Regulation (2018) stating that ‘residents or property owners have a responsibility to not breed mosquitoes on a private property’ [40, 41]. In the interviews, one of the EHWs acknowledged that these regulations, including the possibility of being fined, were helpful to motivate some people to clean up their backyards.

*“Well, people do get fined if they have things laying around everywhere you went.”(TSIRC,3)*.

Although property owner’s are responsible for preventing mosquito breeding, when there is an identified mosquito-borne disease outbreak risk, Queensland Health can approve an Authorized Prevention and Control Program (AAEP) to manage this risk [41]. The AAEP uniquely grants authorized personnel to enter private premises without a warrant or occupier consent to conduct vector control [41]. In the interviews, several AAEP staff stated that working within an Authorized Prevention and Control Program influenced the community engagement strategies used, particularly during the *cordon sanitaire* phase on Thursday Island and Horn Island. Although the AAEP still required local council approval, once these approvals were granted, the AAEP staff could enter private properties and conduct source reduction strategies on behalf of residents.

*“When we have processed all the paperwork for the authorized prevention control program that goes into the [news] paper once. Basically for the whole season – each time we come, we don’t have to announce we are coming”. (QH,1)*

This legislative authority also made it quicker to conduct vector control, without the need to actively engage residents.

*“It got to the point where you didn’t want to disturb someone as we knew people were having a sleep in the day, we then just walk in and out”. (QH, 2)*

### Factor two: Cognitive factors – attitude and beliefs influencing community participation approaches

The community participation approaches used by both the AAEP staff and the TSIRC EHWs were influenced by their beliefs and attitudes toward engaging the community. These beliefs and attitudes stemmed from personal experience, community connections, and their views on the role of community in mosquito management. For example, many of the EHWs described having positive experiences with engaging community members in *Aedes* mosquito management, which helped motivate them to involve the community in this work. In addition, most of the EHWs lived on the islands they were working on, so they knew the community and had established rapport and trust. Many of the EHWs also described how they felt the community were largely supportive of being engaged in mosquito management, due to community pride, the outdoor lifestyle and the connectedness that comes with living in a small community.

*“The community help us in cleaning. And if you come to [redacted] Island, you see there’s no dirt anywhere lying around like you would expect in [other] communities.” (TSIRC, 3)*.

“*We spend most of our time on outside fireplace so they can go cook up their fish and stuff. And people just like being outside in the yard, so they’re going to clean up. If they don’t, then you got bothered.” (TSIRC,1)*.

*“I guess, because a lot of the communities are small, so if there ever was an outbreak, then all of us are at risk. Then everyone works together so that doesn’t happen. So because you know, everyone’s family, everyone knows everyone. Because, you know, look after yourself, look after your family, because that’s why we clean up.*” *(TSIRC, 1)*.

Some the EHWs had mixed views on whether community members should take more of an active role in mosquito management, beyond just cleaning up their backyards. For example, one EHW supported the idea of using citizen science approaches to actively involve community members, including children, in broader mosquito management activities.

*“I think it would be good if something happened where they [kids] can be like. junior mosquito management or something.” (TSIRC,1)*.

Conversely another EHW felt that the community should just focus on reducing aquatic habitats in and around their homes.

*“You don’t want to pile too much in the head. You tell them the minimum they need to know. What they need to do as a householder, cleaning the yard, keeping the yard rubbish collection, all that. If they’ve got wrigglers, come and see us…They’ve got other things to do.” (TSIRC,3)*.

When the AAEP was running on the outer islands during Phase One (2005–2009), the attitude of the AAEP staff towards engaging community members and the local EHWs was partly shaped by their experience of being outsiders to the Torres Strait. Most of the AAEP staff acknowledged the importance of engaging with the local EHWs to help familiarize them with the different island communities, and to assist with building community trust and support, particularly given the AAEP was a new program requiring vector control staff to enter people’s properties.

“*You had to because of the cultural differences that you have to work with and respect that.”(QH,3)*.

*“It is very important that we have a local, [it] helps to relay the message to people - what we are about. It gives people the confidence when we are entering properties. This is [the] outer islands mostly.”(QH,1)*.

One of the AAEP staff noted that it was also a community expectation that the EHWs were present when entering people’s properties.

*“The concern was they didn’t want government workers knocking on people’s doors without a local person.” (QH,2)*.

Conversely, one AAEP staff described their experience engaging the EHWs prior to the AAEP, and recalled this to be a challenge, which in turn influenced their views on engaging the EHWs as part of AAEP.

*We tried [to engage the local EHWs] before the Albo [AAEP] program started, we had a bit of dengue on the outer islands. We would get help the first day, often, then sometimes the second day they would say ‘oh I have to do my email’ […]we didn’t get a lot of work done.” (QH,2)*.

When the AAEP shifted focus to *cordon sanitaire* on Thursday Island and Horn Island (Phase Two) the community participation strategies also changed, with notably less engagement with the EHWs and community members. Some AAEP staff felt it was more efficient for the AAEP staff to conduct vector control on their own.

*“Once the TI [Thursday Island] program started we realized we were better off on our own.”(QH,2)*.

Another reason cited for the change in community participation strategies was the AAEP staff’s belief that community members on Thursday Island were not interested in mosquito management and were happy for government agencies to do this work for them.

*“Basically TI [Thursday Island] is like a suburb in Cairns in many, many ways but the attitude’s not the same. It’s not one of their [community] priorities put it that way. […] TI [Thursday Island] is like a government island, a hub.” (QH,2)*.

*“On TI [Thursday Island] it’s big, everyone does their own thing. They know us, they see us around. It’s a little bit different from the outer islands.” (QH, 4)*.

Despite less engagement with the community and EHWs, the DART continued to engage with local elders believing it was important to gain their support for the AAEP, particularly on Horn Island. One of the AAEP staff described the Horn Island community as being different to Thursday Island, with more people of Aboriginal heritage, which meant different cultural protocols needed to be followed.

*“When we were on Horn Island, there were tribal elders set up but basically you had to go run things past them - ask their permission. Certain areas that you can’t go into without their permission.[…]There are certain people within the community that like to be shown a little bit more respect going onto their property, showing a bit of courtesy.” (QH, 3)*.

### Factor three: Resource factors influencing community participation approaches

Human resources, time pressures and physical resource constraints influenced the community participation approaches used in *Aedes* mosquito management in the Torres Strait. Although most of the EHWs felt they had sufficient physical and human resources to engage the community on the outer islands, there were mixed views regarding whose responsibility it was to support community members in getting rid of large potential mosquito breeding sites such as unwanted cars and white goods. For example, in the past Queensland Health had implemented one-off strategies to remove large items from some of these islands. One Queensland Health staff felt that resources to support the removal of unwanted items off the islands was a local government issue that should be dealt with at a local level. They also described the changes that had occurred since the 2008 Local Government Reform, when previous to this, there had been support for assisting community members in dengue fever prevention. They also felt the reform had resulted in less resources given they were now being pooled under one council.

*“…the resources were there on the ground by local government councils here to remove waste, big macro waste like your boats, your cars, your tanks, whatever…But now, you have minimum resources because 15 communities have got to compete for that one set of resources.” (QH,5)*.

For the AAEP, time constraints driven by the fly-in, fly-out operating model influenced the community participation approaches used by the AAEP staff. For example, during Phase One, AAEP staff experienced significant time pressures travelling to multiple islands and liaising with the EHWs, community leaders and community members over a short period of time. These resource and programmatic pressures to be efficient, interacted with the underlying attitudes and beliefs of the AAEP staff (as reported above) that they were better off working independently (rather than with the EHWs and community members) in order to remain focused and efficient.

*“Sure people [EHWs] are doing great things, we’ve seen some great results but that’s not how this program works. You can’t just say I’m sorry I just can’t work for the next five days. Things like holidays, Sorry Business, everything puts a stop. Everyone needs to do what they need to do but the program doesn’t stop for anybody”. (QH,4)*

During Phase Two of the AAEP, although the AAEP staff had less islands to cover, time and efficiency were still important, with additional, more intensive vector control strategies being implemented compared to Phase One. Vector control strategies in Phase Two included harbourage spraying using a tractor-mounted high pressure spray unit, which required specific vector control expertise and a pest control licence. Hence the DART felt there was little point to engaging community members in these strategies.

*“Remember that albopictus is peri-domestic, that’s why we do harbourage spraying. For citizen science…it would be pointless”. (QH,6)*

In the interviews, when the AAEP staff were asked whether the fly-in, fly-out workforce was a cost effective, sustainable mosquito model, most acknowledged that this model was not ideal for promoting long-term, community-based mosquito management. However, one AAEP staff viewed the AAEP funding as small compared to the cost of managing an *Ae. albopictus* mosquito incursion into Australia. There was also the suggestion that this may be the only approach until population replacement or genetic mosquito modification strategies are introduced to the Torres Strait.

*“I guess long term they would look at Wolbachia – that would be the only way forward. If not, it would be crazy to change the program, in the meantime because what we are doing is so effective. Yes, it’s labour intensive but when you look at the big picture in Australia what is [redacted] dollars?” (QH,4)*.

One Queensland Health staff expressed concern around the practicalities of sustaining the fly-in, fly-out model, for the main reason that it was hard to meaningfully engage the community. They suggested that the AAEP should be managed by the Torres Strait community, rather than externally administered, to potentially improve the sustainability of the program.

*“…I disagree with fly in, fly out, because you don’t build a rapport with the community. And if you build a rapport with your community, people need to know that when you walk into our communities, the family and social dynamics are different because you got cultural and traditional dynamics that affect community as well. So what I’d like to see, is community-driven, sustainable workforce… People will do what is driven by the money, but for a sustainable workforce, you’ve got to have it built from a community up the grassroot level because no one knows their community the best like they do”. (QH, 5)*

## Discussion

While community participation is an important part of *Aedes* mosquito management, existing literature suggests a preference for government-led, top-down approaches in high-income countries [13].This research, based on a comprehensive document analysis and key informant interviews, offers insight into the community participation approaches used in the Torres Strait, and sheds light on the key factors that shaped their selection.

In the first instance, our research revealed a range of different community participation approaches used in *Aedes* mosquito management in the Torres Strait. Collectively these approaches align with the five community participation levels (informing, consulting, involving, collaborating, and empowering) described by well-known frameworks such as the IAP2 Public Participation Spectrum© [31]. For example, Phase One of the AAEP engagement on the outer islands used informing and consulting approaches, to gain support for the program, to raise awareness of dengue fever and to encourage source reduction. Phase Two engagement on Thursday Island and Horn Island focused primarily on top-down, informing approaches, with the purpose of letting the community know when the AAEP were operating. Similarly, the TSIRC Environmental Health Program used informing and consulting approaches to engage the community in promoting source reduction efforts. The overall focus on more top-down, informing and consulting approaches, aligns with strategies commonly used to engage communities in dengue fever prevention in high-income countries [29].

Additionally, this research found examples of approaches that support community ownership and empowerment. Laverack (2001) describes seven key domains of empowerment including linking with others, women’s involvement, local assessment of problems, individual and group participation, local resource mobilisation, developing local leadership and asking why [32].The TSIRC Environmental Health Program demonstrated some of these domains in their operations, such as developing local leadership through the local environmental health workers, using local resources and knowledge, and encouraging individual and group participation. For example, although the EHWs were managed under one local government council, they had autonomy to implement mosquito management strategies as they saw fit on each island. Self-determination and empowerment are key drivers for Aboriginal and Torres Strait Islander communities in gaining control of their lives, therefore the local leadership and community participation elements of this program are noted as being important [42, 43].

We also explored the key factors influencing the choice of these community participation approaches. We found a complex interplay of regulatory, cognitive, and resourcing factors shaping these choices. For example, when the AAEP shifted focus to Thursday Island and Horn Island (Phase Two), the transition to a more top-down approach was partially a product of the AAEP teams’ own attitudes and beliefs that intensive vector control operations would be more efficient and effective, without the need to actively engage the EHWs and the community. These beliefs were supported by legislated power under the Queensland Public Health Act (2005) for the AAEP to enter private properties and conduct vector control on behalf of residents [41]. In addition, high stakes related to the public health imperative that underpinned program funding; that is, to prevent the *Ae. albopictus* from establishing on mainland Australia, directed staff away from more time-consuming community engagement approaches [16].

Although the AAEP has so far been successful in suppressing the *Ae. albopictus* to almost undetectable levels on Horn Island and Thursday Island, [16] our research highlights concerns linked to the reliance on a fly-in fly-out workforce and the top-down nature of operations which has resulted in limited engagement with the local vector control workforce and community members. Top-down approaches can lead to government dependence, and as highlighted in this research, require extensive resourcing commitments [44].

In consideration of the AAEP in its current operating form, we identify opportunities for community members and local environmental health staff to play a more active role in this program. For example, a growing number of citizen science projects in Australia, and globally, involve community members in *Aedes* mosquito surveillance [45]. As ‘sweep netting’ is one of the primary methods of assessing *Ae. albopictus* abundance on Thursday Island and Horn Island, [16] community members or local environmental health staff could lead or support the implementation of this strategy, to help build local vector surveillance capacity and create a sense of ownership. There may be similar opportunities in the outer islands to build the capacity of the local EHWs or community volunteers. Other invasive species eradication programs in Queensland, such as the Yellow Crazy Ant Eradication Program use a community-run, volunteer taskforce to help with monitoring activities, completeing surveys and assisting with treatment. A similar approach could be used to support vector surveillance and control efforts in the outer islands [46].

Furthermore, engaging community members in source reduction should continue to be a priority, particularly on Thursday Island, where *Ae. aegypti* is in abundance [16]. With the current focus on controlling *Ae. albopictus* using specific measures such as harbourage spraying, coupled with the limited engagement of community members in source reduction, the presence of *Ae. aegypti* could increase disease risk on Thursday Island [47] .

### Further research

Our research revealed the different community participation approaches used in *Aedes* mosquito management in the Torres Strait, and the factors influencing the choice of these approaches. We did not assess the effectiveness of these strategies and therefore it would be useful for future research to focus on understanding which strategies work best to support effective and sustainable *Aedes* management efforts. As an example, evaluating the impact of existing approaches such as the community clean-up events on behaviour change, or larvae and pupae density, could contribute to building the evidence base for using this strategy in *Aedes* management in this region, as well as in other tropical regions.

In addition, understanding community member’s perspectives on the current and future engagement strategies used in the Torres Strait would be useful to inform future engagement in this area. In light of the current AAEP fly-in, fly-out workforce model, it would also be pertinent to continue to explore the perspectives of the Torres Strait environmental health workforce on how they view sustainable, community-led *Aedes* mosquito management, particularly on Thursday Island and Horn Island.

## Conclusions

This study offers valuable insights into the community participation strategies used in *Aedes* mosquito management within the Torres Strait. Traditionally, high-income countries have favoured government-led, top-down approaches for engaging communities in mosquito management. Our research uncovers an array of approaches in the Torres Strait, encompassing both top-down and bottom-up methods. The decision-making behind these strategies was found to be influenced by a combination of regulatory, cognitive, and resource-related factors. These findings not only shed light on the rationale for why top-down approaches prevail in *Aedes* mosquito management within high-income countries, but also describe opportunities to enhance community participation in *Aedes* mosquito management in the Torres Strait. These opportunities could also be considered in other similar tropical regions experiencing *Aedes-*borne risk.

## Data Availability

James Cook University has a managed access system for data sharing that respects legal and ethical obligations to study participants to collect, manage and protect their data. Summarized non-identified data supporting the conclusions of this article can be made available from the corresponding author (TA) upon reasonable request.

## Abbreviations

TSIRC: Torres Strait Island Regional Council
EHW: Environmental Health Worker
AAEP: *Ae. albopictus* Eradication/Control Program
